# An Optimized SPME-GC-MS Method for Volatile Metabolite Profiling of Different Alfalfa (*Medicago sativa* L.) Tissues

**DOI:** 10.3390/molecules26216473

**Published:** 2021-10-27

**Authors:** Dong-Sik Yang, Zhentian Lei, Mohamed Bedair, Lloyd W. Sumner

**Affiliations:** 1 The Samuel Roberts Noble Foundation, 2510 Sam Noble Parkway, Ardmore, OK 73401, USA; dongsiki@hanmail.net; 2 Metabolomics Center, Department of Biochemistry, University of Missouri, Columbia, MO 65211, USA; leiz@missouri.edu; 3 Bayer CropScience, 700 Chesterfield Parkway, West Chesterfield, MO 63017, USA; mohamed.bedair@bayer.com

**Keywords:** *Medicago sativa*, alfalfa, SPME, GC-MS, volatiles

## Abstract

Solid-phase microextraction (SPME) was coupled to gas chromatography mass spectrometry (GC-MS) and a method optimized to quantitatively and qualitatively measure a large array of volatile metabolites in alfalfa glandular trichomes isolated from stems, trichome-free stems, and leaves as part of a non-targeted metabolomics approach. Major SPME extraction parameters optimized included SPME fiber composition, extraction temperature, and extraction time. The optimized SPME method provided the most chemically diverse coverage of alfalfa volatile and semi-volatile metabolites using a DVB/CAR/PDMS fiber, extraction temperature of 60 °C, and an extraction time of 20 min. Alfalfa SPME-GC-MS profiles were processed using automated peak deconvolution and identification (AMDIS) and quantitative data extraction software (MET-IDEA). A total of 87 trichome, 59 stem, and 99 leaf volatile metabolites were detected after background subtraction which removed contaminants present in ambient air and associated with the fibers and NaOH/EDTA buffer solution containing CaCl_2_. Thirty-seven volatile metabolites were detected in all samples, while 15 volatile metabolites were uniquely detected only in glandular trichomes, 9 only in stems, and 33 specifically in leaves as tissue specific volatile metabolites. Hierarchical cluster analysis (HCA) and principal component analysis (PCA) of glandular trichomes, stems, and leaves showed that the volatile metabolic profiles obtained from the optimized SPME-GC-MS method clearly differentiated the three tissues (glandular trichomes, stems, and leaves), and the biochemical basis for this differentiation is discussed. Although optimized using plant tissues, the method can be applied to other types of samples including fruits and other foods.

## 1. Introduction

Solid-phase microextraction (SPME) is a gas-phase, absorptive extraction method used for the isolation of volatile organic compounds with detection limits in the range of parts per billion by volume through static headspace sampling and GC-MS analyses. SPME has been widely used for plant [1], fruit [2,3], beverage [4,5,6], and meat [7,8] volatile analyses because it is simple, automatable, highly sensitive, and rapid.

**Figure 1:**
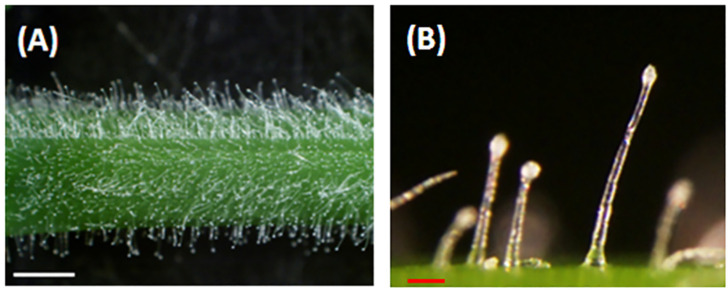
Alfalfa glandular trichomes. (**A**). Glandular trichomes on an alfalfa (FSG 420LH) stem. (**B**). Magnified view of glandular trichomes showing stalks and secretary heads. White bar = 1 mm, red bar = 0.1 mm.

Alfalfa (*Medicago sativa* L.) is a perennial flowering legume grown in many parts of the world that serves as a premium forage used to feed animals. Previous studies have demonstrated that alfalfa varieties with resistance to potato leafhopper (PLH) have a high density of glandular trichomes on their stems [9,10]. Plant glandular trichomes are epidermal appendages with a stalk and secretary head (Figure 1). These organs are known to synthesize, accumulate, and secrete volatile metabolites to protect themselves against pathogens and herbivores [11,12]. A previous report suggested that volatile metabolites released by glandular trichomes in alfalfa might play important roles as repellents, deterrent, or fumigants to PLH [13,14]. However, there is minimal information on the vast array of volatiles produced by alfalfa glandular trichomes. Volatile metabolites in leaves and flowers of alfalfa were analyzed using dynamic headspace analyses with Tenax trapping, and a modest number volatile metabolites were detected (16 leaf and 33 floral) using a vacuum steam distillation method [15,16].

Volatiles emitted from fruits and plants are typically very diverse, including alkanes, alcohols, aldehydes, ketones, terpenoids, and esters. The absorption efficiency of these compounds depends upon the SPME fiber type, extraction temperature, and time. Here, we report the optimization of a SPME extraction procedure using alfalfa leaves as samples. The effects of three different fibers, extraction temperature, and extraction time on the volatile extraction efficiency were investigated and optimized. These specific parameters were investigated because they critically affect the equilibrium of volatiles between the fiber, headspace, and sample [17,18,19,20,21]. The optimized method was then applied to the large-scale, non-targeted profiling of volatile metabolites emitted from glandular trichomes isolated from stems, stems free of trichomes (i.e., stem after isolation of trichomes), and leaves of alfalfa. A substantial number of volatile metabolites were detected and identified, and the efficiency of the optimized SPME-GC-MS method was validated using multivariate analyses including hierarchical cluster analysis (HCA) and principal component analysis (PCA).

## 2. Results and Discussion

### 2.1. SPME Extraction Parameters

An optimized SPME method was developed for the analysis of a broad range of volatile metabolites from fresh alfalfa plants using a non-targeted metabolomics approach. Parameters investigated included fiber composition, extraction temperature, and extraction time. These parameters were compared and optimized on the basis of both of the quantitative and qualitative composition from the large-scale volatile metabolic profiling associated with the various tested conditions, whereas many studies have considered only the total amount of detected metabolites or the detected number of targeted metabolites during determination of optimal SPME extraction parameters [18,19,20,22,23].

### 2.2. Fiber Composition

The SPME fiber composition and selectivity were studied. Three types of fibers (PDMS, CAR/PDMS, and DVB/CAR/PDMS) with different thickness and polarity were studied. These fibers were evaluated for their diverse coverage of volatile metabolites emitted from alfalfa based on prior SPME studies of plant volatile analyses [24,25,26]. The extraction of volatile metabolites emitted from alfalfa leaves was first performed with various fibers at 50 °C for 20 min. Table 1 shows that the bipolar CAR/PDMS fiber (99 volatile metabolites) and DVB/CAR/PDMS fiber (97 metabolites) extracted twice the number of volatile metabolites as did the non-polar PDMS fiber (48 metabolites).

These results demonstrate that the bipolar fibers are qualitatively better for isolating a larger diversity of volatile metabolites emitted from alfalfa than the non-polar fiber. Nineteen of the volatile metabolites detected in the PDMS fiber were tentatively identified, while 32 and 38 of the volatiles detected in the CAR/PDMS and DVB/CAR/PDMS fibers, respectively, were putatively identified. These included the quantitative major volatile metabolites emitted from alfalfa leaves and were classified into five functional classes (i.e., alcohols, aldehydes, ketones, terpenoids, and others) (Table 1). The CAR/PDMS and DVB/CAR/PDMS fibers had very similar extraction efficiencies for alcohols, aldehydes, ketones, and others (e.g., 2-methylfuran, 2-ethylfuran, 3-ethylthiophene, 5-ethyl-2(5*H*)-furanone, (*E*)-3-hexen-1-ol acetate, ionene, β-Ionone). This included 5 volatile alcohols for each of the CAR/PDMS and DVB/CAR/PMDS fibers respectively; 13 and 14 volatile aldehydes; 6 and 5 volatile ketones; and 6 and 6 volatile others. However, the CAR/PDMS fiber failed to extract 6 of the 8 terpenoid volatile metabolites detected with the DVB/CAR/PDMS fiber [i.e., (+)-(*R*)-limonene, *cis*-linalool oxide, β-linalool, *p*-menth-1-en-4-ol, α-terpinol, and β-damascenone], indicating that the CAR/PDMS fiber is more limited in its ability to extract non-polar terpenoid volatile metabolites. Terpenoids serve important physiological and ecological roles as attractants, repellents, anti-feedants, toxins, or antibiotics in most plant species, and have been used by human as pharmaceuticals, flavors, fragrances, food supplements, and natural pesticides [27,28].

The number of volatile metabolites extracted in the CAR/PDMS fiber and the DVB/CAR/PDMS fiber were not statistically different; however, the DVB/CAR/PDMS fiber was able to extract more chemically diverse metabolites, including alcohols, aldehydes, ketones, terpenoids, and others. Therefore, the DVB/CAR/PDMS fiber was selected for further optimization due to its greater ability to provide a more comprehensive chemical profile of alfalfa volatiles.

### 2.3. Extraction Temperature

**Figure 2:**
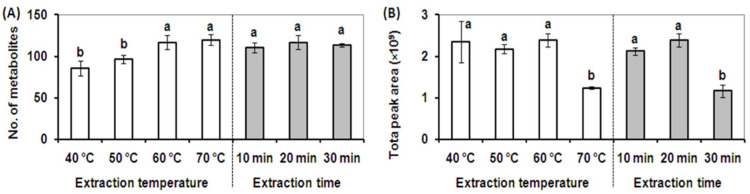
Effect of temperature and extraction time on SPME extraction performance. (**A**). The total number of volatile metabolites detected at different extraction temperatures for 20 min (white column) and different extraction times at 60 °C using a DVB/CAR/PDMS SPME fiber (gray column). (**B**). The total peak area of metabolites detected at different extraction temperatures for 20 min (white column), and different extraction times at 60 °C using a DVB/CAR/PDMS SPME fiber (gray column). Vertical bars with the same letter are not significant; however, those indicated with a different letter are significant according to the Duncan’s multiple range test (*p* < 0.05). These results illustrate that the broadest cross-section of volatile and semi-volatile metabolites was detected using an extraction temperature of 60 °C and an extraction time of 20 min.

The extraction temperature for the DVB/CAR/PDMS fiber was optimized using an extraction time of 20 min. Four different extraction temperatures of 40, 50, 60, and 70 °C were investigated. As shown in Figure 2A, the metabolite profiles obtained using extraction temperatures of 60 and 70 °C contained the highest number of metabolites, and elevated temperatures increased the number of detected metabolites by 121% and 124% respectively compared to an extraction temperature of 50 °C. However, the total peak area of the metabolite profiles obtained at the extraction temperature of 60 °C was twice that obtained using an extraction temperature of 70 °C. Further, the broadest cross-section of volatile and semi-volatile metabolites was detected using an extraction temperature of 60 °C (Figure 2B). A temperature greater than 60 °C dramatically decreased the quantitative extraction efficiency for volatile metabolites in alfalfa due to a decrease in the partition coefficients and decreased extraction by the SPME fiber [18]. The extraction temperature plays an important role in transferring analytes from the solution to the headspace and from the headspace onto the fiber by affecting the diffusion rate of analytes and vapor pressure of the analytes in the sample [18,23]. An optimized extraction temperature of 60 °C was selected for the optimized SPME extraction method.

### 2.4. Extraction Time

Three different extraction times (10, 20, and 30 min) were then tested at 60 °C using the DVB/CAR/PDMS fiber. As shown in Figure 2A, the extraction times tested showed a negligible effect on the number of metabolites detected, however, an extraction time of 20 min led to the highest total peak area. The total peak areas in extractions longer than 20 min were dramatically decreased due to the partial desorption of some volatile metabolites from the SPME fiber through a competition phenomenon which is based upon the equilibrium between analytes and the fiber coating (Figure 2B) [18]. The competition phenomenon results in different quantitative recoveries for different extraction times, whereas fiber composition and extraction temperature contributed to the qualitative composition as well as the quantitative recovery of volatile metabolites in alfalfa plants. A final extraction time of 20 min was selected due to better metabolite adsorption efficiency and quantitative recovery using the DVB/CAR/PDMS fiber.

The optimized SPME extraction method for the large-scale volatile metabolic profiling of alfalfa consisted of a DVB/CAR/PDMS fiber, an extraction temperature of 60 °C, and an extraction time of 20 min. The absorptive composition of the SPME fiber was the most important parameter for optimization as it had the greatest effect on both the qualitative and quantitative volatile profiles. The extraction temperature and time were also important parameters, because they have a substantial impact on the transfer and equilibrium between the sample analyte matrix and the SPME fiber [19,29].

### 2.5. Large-Scale Profiling of Alfalfa Volatile Metabolites

The optimized SPME-GC-MS method was used for nontargeted volatile metabolic profiling of alfalfa glandular trichomes from stems, trichome-free stems, and leaves. The analyses were performed using four replicates and the optimized SPME-GC-MS method. Data processing was performed using Automated Mass spectral Deconvolution and Identification System (AMDIS) software [30]. A total of 87 volatile metabolites were detected in glandular trichomes, 59 in stems, and 99 in leaves (Supplemental Table S1) after background subtraction to remove contaminants present in ambient air and associated with the NaOH/EDTA buffer solution containing CaCl_2_. Thirty-seven volatile metabolites were consistently detected in all samples while another 15 volatile metabolites were uniquely detected in glandular trichomes, 9 only in stems, and 33 specifically in leaves. Confident identification of 36 glandular trichomes, 31 stems, and 41 leaves volatiles was accomplished through mass spectral matching of experimental data with the NIST08/Wiley7 mass spectral library, retention indices (RIs) from the literature, mass spectra of authentic compounds, and RIs of authentic compounds consistent with Metabolomics Standards Initiative [31]. Additional metabolites were only tentatively identified due to the absence of authentic standards or difficulty in measuring RIs of very early eluting compounds. Tentatively identified early-eluting metabolites without RIs included: sulfur dioxide, acetaldehyde, methanethiol, ethyl alcohol, 2-propenal, and 2-propanone (Supplemental Table S1).

**Figure 3:**
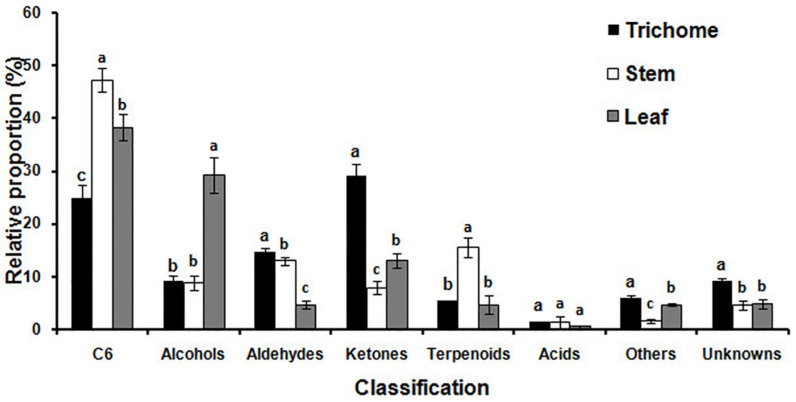
The relative proportion of specific chemical classes of volatile metabolites emitted from glandular trichomes isolated from stems, trichome-free stems, and leaves were significantly different. Vertical bars with the same letter are not significant; however, those indicated with different letters are significant according to the Duncan’s multiple range test (*p* < 0.05).

MET-IDEA software was used for quantitative data extraction using the most abundant fragment ion from the multiple model ions reported by AMDIS [32,33]. The relative concentration (μg g^−1^ fr. wt) of each metabolite was calculated by dividing the extracted value of each metabolite by the extracted value of 3-pentanol (internal standard) and fresh weight of the sample. Reproducibility of the analytical process was good, as demonstrated by the small relative standard deviations (RSD) observed for four replicates of the internal standard equal to 7.1% for glandular trichomes, 10.3% for stems and 4.4% for leaves. The total relative concentrations of volatile metabolites in glandular trichomes, stems, and leaves were 0.66, 0.87, and 10.72 mg g^−1^ fr. wt, respectively. The total relative concentration in leaves was 16.2 and 12.4 times higher than that in glandular trichomes and stems, indicating that alfalfa leaves are quantitatively the main source of volatile metabolites. The relative proportion of the primary classes of volatile metabolites in glandular trichomes, stems, and leaves were significantly different from other plant tissues as shown in Figure 3. Ketones (29.1%), C6 metabolites (24.9%), and aldehydes (14.7%) were quantitatively the most abundant metabolites in glandular trichomes. The major metabolites in stems were C6 metabolites (47.3%), terpenoids (15.5%), and aldehydes (13.0%). The quantitatively most abundant volatile metabolites in leaves were C6 metabolites (38.4%), alcohols (29.2%), and ketones (13.1%).

The C6 metabolites ((*Z*)-3-hexenal, hexanal, 2-hexyn-1-ol, (*E*)-2-hexenal, 1-hexanol, (*E*,*E*)-2,4-hexadienal, and (*Z*)-3-hexenyl acetate) are the main contributors to the green sensory characteristics of many plants as green leaf volatiles (GLV), and this was the largest class of volatile metabolites in alfalfa stems and leaves [34]. GLVs including alcohols, aldehydes, and esters of C6 metabolites are synthesized from C18 unsaturated fatty acids (linoleic and linolenic acid) via the lipoxygenase activity. The aldehydes of the C6 metabolites can be converted to their isomers by alkenal isomerases, or they can be reduced to alcohols and esters by alcohol dehydrogenases and alcohol acetyl transferases, respectively [35]. (*E*)-2-Hexenal was the most abundant metabolite of the C6 family of metabolites in stems (270.85 μg g^−1^ fr. wt) and leaves (2310.32 μg g^−1^ fr. wt). The physiological roles of (*E*)-2-hexenal include high antibacterial activity against Botrytis cinerea in Arabidopsis. It also inhibits root elongation in Arabidopsis, and triggers signaling within and between plants to emit volatile compounds in tomato [36,37,38]. (*Z*)-3-Hexenyl acetate, which is released upon mechanical wounding of the leaves, was present only in leaves (23.29 μg g^−1^ fr. wt) [39]. The relative proportion of alcohols in leaves was significantly more than three times higher than in glandular trichomes or stems. The most abundant alcohol in leaves was 1-octen-3-ol, which is known as one of the major aroma-active compounds and an insect attractant.

The relative proportion of aldehydes in glandular trichomes and stems was similar, and the major aldehyde metabolites were nonanal and decanal in these two samples. However, the major aldehyde in leaves was (*E*)-2-pentenal, followed by octanal, (*E*,*E*)-2,4-heptadienal, and (*E*)-2-heptenal. The relative proportion of ketones in glandular trichomes was significantly higher than in stems and leaves, 3.2 and 2.2 times higher respectively, and 2-propanone was qualitatively the primary ketone in glandular trichomes, contributing 25.1% of the total trichome ketones.

Terpenoids made up 15.5% of total relative volatiles in stems, but only 5.4 and 4.7% in glandular trichomes and leaves, respectively. α-Terpinol was the most abundant metabolite in glandular trichomes, followed by β-linalool, *cis*-linalool oxide, isoprene, *p*-cymene, D-limonene, and *p*-menth-1-en-4-ol. However, the most abundant metabolites in stems, in decreasing order of their relative concentrations, were α-terpinol, β-linalool, *cis*-linalool oxide, ocimenol, and myrcene. The most abundant metabolites in leaves, in decreasing order of relative concentration, were β-linalool, α-terpinol, *cis*-linalool oxide, (*E*,*E*)-2,6-dimethyl-1,3,5,7-octatetraene, *p*-menth-1-en-4-ol, and isoprene. The different metabolic profiles in glandular trichomes, stems, and leaves revealed that biosynthesis and emission of volatile metabolites varied substantially in different tissues.

### 2.6. Multivariate Analysis

Multivariate statistical analyses including HCA and PCA allow for the classification and visualization of volatile SPME-GC-MS data. They also enable the determination of specific variables that contribute to the difference among the tissues based upon the mass spectrometry data [40]. For example, multivariate analyses of volatile metabolites emitted from ripe tomato fruits allowed distinct separation of structurally related metabolites derived from the same biochemical precursors, and treatments of wounded leaves from Nicotiana attenuate were separated based upon their volatile composition [41,42].

**Figure 4:**
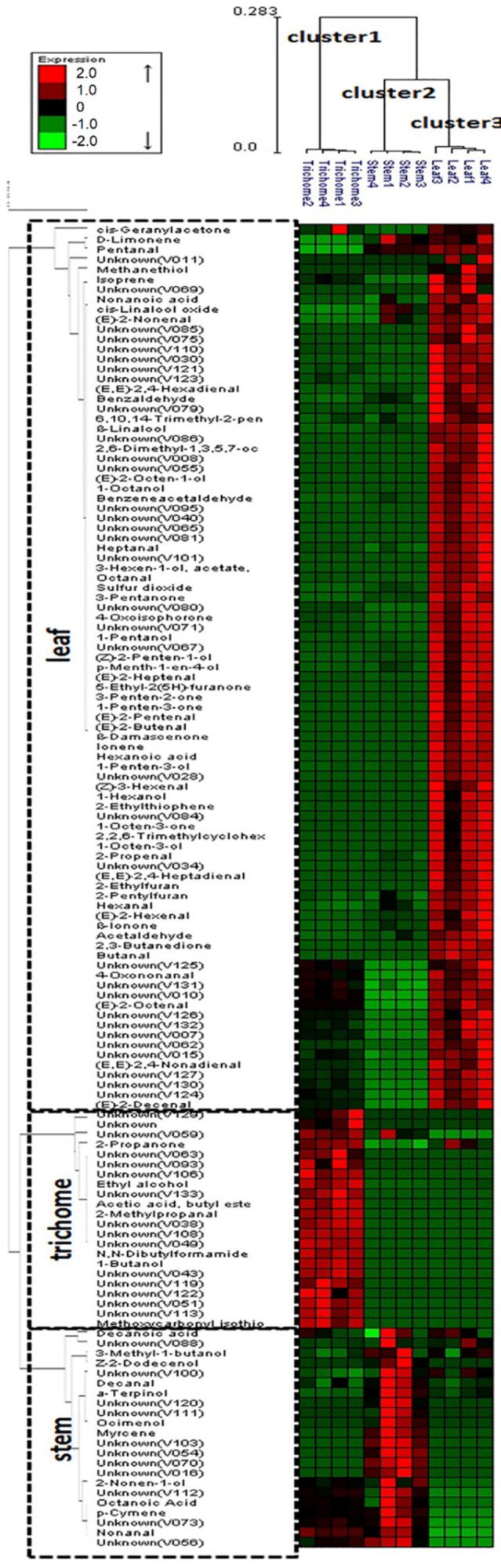
Dendrogram obtained from the hierarchical cluster analysis of plant tissue SPE-GC-MS data (i.e., glandular trichomes isolated from stems, trichome-free stems, and leaves of alfalfa plants) and 133 volatile metabolites using Expander package (ver. 6). The dendrogram provides distinct visualization of the differences in volatile metabolites emitted from the various plant tissues: cluster 1 (glandular trichome), cluster 2 (stem), and cluster 3 (leaf). The metabolites contributing to the separation of glandular trichome, stem, and leaf are also shown in the dendrogram.

HCA was used in this study to differentiate volatile profiles from alfalfa trichomes, stems, and leaves. HCA was performed using the average linkage method and the quantitative data for 133 volatile metabolites. The HCA dendrogram allowed distinct visualization of three different groups (Figure 4): cluster 1 (glandular trichome), cluster 2 (stem), and cluster 3 (leaf). In addition, the dendrogram identified specific metabolites contributing to the separation of glandular trichome, stem, and leaf volatile profiles. The level of precision of the analytical method was indicated by the correct classification of four biological replicates among samples and the precise separation of the individual samples.

**Figure 5:**
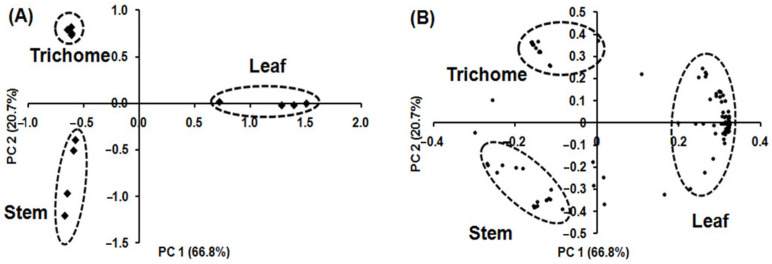
PCA biplot of volatile metabolites originating from glandular trichomes isolated from stems, trichome-free stems, and leaves of alfalfa plants on the basis of 133 volatile metabolites (*n* = 4); (**A**) score plot showing separation of glandular trichomes, stems, and leaves tissue; (**B**) loading plot showing distribution of 133 volatile metabolites. The score plot illustrates clear differentiation between the volatile metabolic profiles of glandular trichomes, stems, and leaves and the loading plot indicates the contributions of independent volatile metabolites on the separations in the PCA score plot.

PCA was applied using the relative concentration of volatile metabolites in each sample to determine which volatile metabolites had the highest loadings and contributed the most to differences in volatile metabolic profiles among the samples (Figure 5). Figure 5A shows the PCA pattern for the different alfalfa tissues, confirming three clusters similar to those obtained by HCA. Volatile metabolic profiles in glandular trichome, stem, and leaf were clearly separated, accounting for 87.5% of the total variance by combining the first (PC 1) and second (PC 2) principal components. PC 1 accounts for 66.8% of the total variance and represents the difference in the volatile metabolic profiling of leaf from that of glandular trichome and stem. PC 2 accounts for 20.7% of the total variance and represents the difference in the volatile metabolic profile of glandular trichome and stem. Figure 5B and Supplemental Table S2 list the 21, 17, and 87 independent volatile metabolites contributing to the separation of glandular trichome, stem, and leaf, respectively.

The major metabolites that influenced the volatile metabolic profile of glandular trichomes, in decreasing order of contribution, were 2-propanone (6; numbers correspond to that in Table S2), ethyl alcohol (4), unknown (108), 1-butanol (18), unknown (43), 2-methylpropanal (14), unknown (38), *N*,*N*-dibutylformamide (107), unknown (49), butyl acetate (33), unknowns (133, 51, 106, 113), methoxycarbonyl isothiocyanate (35), and other unknown/unidentified metabolites (119, 63, 122, 93, 116, 129). The primary metabolites that influenced the volatile metabolic profile of stem, in decreasing order of contribution, were α-terpinol (96), unknowns (103, 54, 70, 52, 16, 120), ocimenol (91), decanal (97), unknown (111), and (*Z*)-2-dodecenol (115). The major metabolites influenced in the volatile metabolic profile of leaf, in decreasing order of contribution, were unknowns (65, 40, 28), 1-pentanol (27), unknown (95), 1-penten-3-ol (19), unknown (81), (*Z*)-3-hexenyl acetate (57), octanal (58), (*Z*)-2-penten-1-ol (29), unknown (71), (*E*)-2-butenal (17), benzeneacetaldehyde (72), unknown (114), 2-ethylfuran (23), ionene (99), hexanoic acid (47), unknown (67), 1-penten-3-one (20), sulfur dioxide (1), 4-oxoisphorone (87), unknown (101), (*E*)-2-octen-1-ol (76), 1-octanol (77), unknowns (80, 34), (*E*)-2-pentenal (26), heptanal (41), (*E*,*E*)-2,4-heptadienal (61), (*E*)-2-heptenal (44), 5-ethyl-2-(5*H*)-furanone (45), and 2-ethylthiophene (39). These metabolites can potentially be used as volatile fingerprints to characterize each tissue.

For example, butyl acetate which critically contributes to ‘Gala’ apple aroma, was present in only trichome tissue [43], while α-terpinol which refers to fragrant, floral, and lilac-like in apricot, had a greater concentration in stem tissue [44]. (*Z*)-3-Hexenyl acetate and 2-ethylfuran were unique metabolites in leaf tissue. (*Z*)-3-Hexenyl acetate plays an important ecological role in airborne wound signaling in plants [45] and 2-ethylfuran is an indicator for the extent of lipid oxidation [46]. There are also greater concentration of C5 metabolites [1-pentanol, 1-penten-3-ol, (*Z*)-2-penten-1-ol, (*E*)-2-pentenal and 1-penten-3-one] in leaf. These are known to be released by wounded leaves of various plants [47].

Alfalfa volatiles have been analyzed before using a number of different techniques to study responses of the plant to pathogens, but these have not used SPME, to the best of our knowledge. Using a vacuum steam distillation method, 16 volatiles, mainly straight chain alcohols, aldehydes, and esters, were identified from leaves of the glandular-haired *M. sativa* G98A and the nongranular-haired *M. sativa* Ranger [15]. A push–pull head-space volatile collection system was also used but identified even fewer volatiles from the intact whole *M. sativa* G98A and Ranger plants [15]. The significantly lower number of volatiles identified using vacuum steam distillation and push–pull methods were probably due to the lack of the volatile enrichment mechanism typically seen SPME methods. This could result if volatiles of low abundances were not detected. For example, all the terpenoids detected in this manuscript were not found in their report [15]. In a different study of *M. sativa* volatiles, 96 volatile compounds were detected from four alfalfa cultivars and three wild *M. sativa* species using a supercritical fluid extraction (SFE) with CO_2_ as supercritical fluid [48]. This number is close to what we detected from *M. sativa* leaves using the optimized SPME method (i.e., 99 volatiles detected). However, SFE is more costly and requires specialized equipment. It is also not considered as an optimal method to study volatile profiles as it does not sample volatiles in the head space emitted by samples. Instead, it extracts compounds, including both volatile and nonvolatile compounds, from the samples using supercritical CO_2_ fluid as extraction solvents. It is not surprising that different metabolite profiles were obtained using SPME and SFE methods. Indeed, a comparison of the volatiles detected revealed that most of the volatiles detected using SFE had larger molecular weights. Some of the low molecular weight volatile compounds that have higher volatility were missing. For example, butenal and propenal that were detected using SPME method were not detected using SFE, suggesting that these low molecular weight volatiles (i.e., compounds of higher volatility) were lost or evaporated when the supercritical CO_2_ fluid was decompressed into gaseous CO_2_. Overall, the optimized SPME method reported here provided a better coverage of alfalfa volatiles.

## 3. Materials and Methods

### 3.1. Chemicals

Authentic standards were co-analyzed and used for confident metabolite identifications [31]. Authentic standards used were 1-butanol, 1-pentanol, 1-hexanol, 2-pentylfuran, (*Z*)-3-hexenyl acetate, (*E*,*E*)-2,4-heptadienal, (*E*)-2-octen-1-ol, β-linalool, nonanoic acid (TCI America, Portland, OR, USA); decanal, decanoic acid, β-ionone, 3-pentanone, butyl acetate, benzaldehyde (Sigma-Aldrich Inc., St. Louis, MO, USA); 2,3-butanedione, 1-penten-3-ol, hexanal, myrcene, *p*-cymene, D-limonene, 1-octanol (Fluka Chemical Co., Milwaukee, WI, USA); isoprene, 1-penten-3-one, pentanal, 2-ethylfuran, 3-methyl-1-butanol, (*Z*)-2-penten-1-ol, (*E*)-2-hexenal, 2-ethylthiophene, heptanal, (*E*,*E*)-2,4-hexadienal, (*E*)-2-heptenal, hexanoic acid, 1-octen-3-ol, octanal, benzeneacetaldehyde, (*E*)-2-octenal, nonanal, 4-oxoisophorone, (*E*)-2-nonenal, octanoic acid, 2-nonen-1-ol, (*E*,*E*)-2,4-nonadienal, (*E*)-2-decenal, *N*,*N*-dibutylformamide (Aldrich Chemical Co., Milwaukee, WI, USA); *p*-menth-1-en-4-ol (Wako Chemical Co., Richmond, VA, USA); and 1-octen-3-one (Alfa Aesar, Ward Hill, MA, USA). 3-Pentanol (Fluka Chemical Co.) was used as an internal standard. The internal standard was chosen due to its stability, reproducibility, and absence in alfalfa plants.

### 3.2. Plant Materials

Alfalfa plants (FSG 420LH) with a high density of glandular trichomes on the stem and resistance to PLH were provided by Forage Genetics International (West Salem, WI, USA). The plants were grown in a greenhouse using 3.8 l pots containing Metro-Mix 830 (Sun Gro, Bellevue, WA, USA) and an 18 h-light/25 °C and 6 h-dark/22 °C photoperiod. Plants were cut back closely to produce vigorous shoots that were then harvested 4 weeks later. Glandular trichomes from stems, trichome-free stems, and leaves were collected for volatile metabolite profiling. Glandular trichomes were isolated from stems starting 5 cm above the crown and approximately 20–30 cm in length. The stems were cut into 2–3 cm long segments and immediately put into a 180 mL glass tube containing liquid nitrogen. The tube was vortexed for 30 s to separate the glandular trichomes from the stems. The resulting trichome-free stem segments were removed, leaving the glandular trichomes in the tube. The collected glandular trichomes, stems, and leaves were stored at −80 °C prior to analysis.

### 3.3. SPME Procedure Optimization

The SPME-GC-MS method was optimized using 500 mg of fresh leaves. The leaves were ground under liquid nitrogen in a mortar and pestle. The ground leaves were placed in 10 mL glass vials (Supelco, Bellefonte, PA, USA) with 1.0 mL of 100 mM EDTA (pH 7.5 with NaOH) containing 5 M CaCl_2_ to inhibit enzyme activity [42] and 10 μL of 3-pentanol (0.82 mg/mL in 1% MeOH in H_2_O) as an internal standard. Nitrogen gas was bubbled through the EDTA and CaCl_2_ solution for 90 min prior to addition to leaf samples, and this process dramatically reduced contaminants in the solution. The vial was tightly sealed with a screw cap containing a PTFE/silicone septum (Supelco, Bellefonte, PA, USA) and then sonicated for 5 min. The vial was placed in a Multipurpose autosampler MPS2 (Gerstel, Munich, Germany) and kept at the same temperature as the extraction during the 20 min equilibration between the solution and the headspace. After the equilibration, the selected SPME fiber was inserted into the headspace of a sample vial for extraction of volatiles.

SPME extraction parameters including fiber composition, extraction temperature and extraction time were optimized to detect the widest quantitative and qualitative array of volatile metabolites in alfalfa plants. All analyses for SPME extraction optimization were performed in triplicate. Three fibers with different polarity were tested: non-polar polydimethylsiloxane (PDMS, 100 μm film thickness, 1 cm fiber length) for extraction of non-polar volatiles; carboxen/polydimethylsiloxane (CAR/PDMS, 75 μm, 1 cm) for extraction of gases and volatiles; and divinylbenzene/carboxen/polydimethylsiloxane (DVB/CAR/PDMS, 50/30 μm, 2 cm) for extraction of volatiles and semi-volatiles with short chains. The extractions were carried out for 20 min at 50 °C. Prior to extraction, each SPME fiber was preconditioned according to the manufacturer’s instructions. Extractions were carried out for 20 min at 40, 50, 60, and 70 °C using DVB/CAR/PDMS fiber to select an optimized extraction temperature, and three time periods (10, 20, and 30 min) were tested at 60 °C using DVB/CAR/PDMS fiber to establish an appropriate extraction time.

### 3.4. Sample Preparation for Volatile Metabolic Profiling

Each tissue was prepared for volatile metabolic profiling by the addition of the EDTA buffer and internal standard, as described above. Headspace volatiles emitted from glandular trichomes (150 mg), trichome-free stems (300 mg), and leaves (300 mg) were extracted using the optimized SPME extraction procedure. The SPME fiber was heated at 250 °C for 20 min to remove carryover between extractions, and four replicates of each tissue were analyzed.

### 3.5. GC-MS Analyses

SPME volatiles were desorbed at 250 °C for 90 s in a splitless injector. Separation of volatiles was achieved with an Agilent 6890/5973 GC-MS (Palo Alto, CA, USA) equipped with a 60 m length, 0.25 mm i.d., 0.25 μm film thickness, fused silica capillary column (DB-5, Agilent). Helium was used as the carrier gas at a flow rate of 1.0 mL/min. The column temperature was held at 40 °C for 2 min and then programmed at 5 °C/min to 250 °C and held for 3 min. MS conditions were as follows: ion source, 200 °C; electron energy, 70 eV; quadrupole temperature, 150 °C; GC-MS interface zone, 280 °C; scan range, 35–350 mass units.

### 3.6. Data Processing, Metabolite Identification and Semi-Quantification

Mass spectrometry data were deconvoluted and analyzed using AMDIS (ver. 2.66, Gaithersburg, MD, USA) (http://www.amdis.net, accessed on 27 October 2021) software for peak determinations and metabolite identification through spectral matching with the NIST 08 and Wiley 7 libraries. The identities of commercially available metabolites were confirmed by comparison of their Kovats’ retention index (RI) and mass spectra of authentic standards. RI values were calculated using a non-polar, DB-5 column and a series of n-hydrocarbons (C5–C33) compared with those reported previously (http://webbook.nist.gov/chemistry/name-ser.html/, last accessed on 25 October 2021). MET-IDEA was used for semi-quantification of each metabolite by extracting representative ion intensity values from the AMDIS output files (*.ELU and *.FIN) for each individual metabolite and then normalization of the relative metabolite abundances to that of the internal standard and sample weight [32,33]. MET-IDEA parameters used included an average width of 0.15 min; minimum peak width of 0.5 times the average peak width; a maximum peak width of 3 times the average; peak start/peak slope of 1.5; an adjusted retention time accuracy of 0.5 times the average peak width; a peak overload factor of 0.75; a mass accuracy of 0.1; a mass window of ±0.02, and a lower mass limit of 0. The semi-quantification of individual volatile metabolites was expressed as 3-pentanol equivalents (response factors were assumed to be 1).

### 3.7. Statistical Data Analysis

Duncan’s multiple-range tests and PCA were conducted using the SAS system for Windows v.8 (SAS Institute, Cary, NC, USA). HCA was performed using the Expander package (ver. 6, Tel Aviv, Israel). The Duncan’s multiple-range tests were performed to select the optimized SPME extraction fiber, temperature, and time. HCA and PCA were carried out to visualize and assess the differences in volatile metabolite profiles from different tissues using the average linkage method and the correlation matrix obtained from data matrix, respectively.

## 4. Conclusions

In this study, an optimized SPME-GC-MS method for volatile metabolic profiling of alfalfa was developed by determining the most suitable extraction fiber, temperature, and time. The optimized SPME-GC-MS method provided large-scale non-targeted profiles of volatile and semi-volatile metabolites emitted from select alfalfa plant organs (i.e., glandular trichomes, stems, and leaves). The accuracy and efficiency of the optimized method was validated, showing a clear differentiation between the volatile metabolites in alfalfa glandular trichomes, stems, and leaves, and the most important contributors to differences in the volatile metabolic profiles were determined. The optimized SPME-GC-MS method will be further incorporated into our metabolomics platform to better understand the physiological and ecological roles of volatile metabolites in alfalfa.

## Figures and Tables

**Table 1:** Volatile metabolites detected in alfalfa leaves using different SPME fibers at 50 °C for 20 min.

RT *^a^*	RI *^b^*	Compound	PDMS	CAR/PDMS	DVB/CAR/PDMS
**Alcohols**					
7.27	680	1-Penten-3-ol	0.39 ± 0.05 *^c^*	N.D.	6.09 ± 1.14
8.57	725	3-Methyl-1-butanol	N.D.	1.12 ± 0.06	N.D.
8.84	734	2-Pentyn-1-ol	N.D.	0.85 ± 0.18	0.22 ± 0.03
9.53	752	(*Z*)-2-Penten-1-ol	N.D.	8.26 ± 0.14	1.56 ± 0.63
12.02	827	2-Hexyn-1-ol	0.49 ± 0.06	N.D.	7.51 ± 0.79
16.59	981	1-Octen-3-ol	55.85 ± 9.49	386.22 ± 13.22	335.81 ± 26.62
19.43	1068	(*E*)-2-Octen-1-ol	N.D.	11.42 ± 8.32	N.D.
**Aldehydes**					
4.75		2-Propenal	N.D.	0.45 ± 0.04	1.10 ± 0.19
5.71	595	Butanal	0.04 ± 0.00	0.34 ± 0.11	0.28 ± 0.06
6.66	645	2-Butenal	N.D.	0.92 ± 0.06	0.47 ± 0.01
7.62	700	Pentanal	N.D.	N.D.	0.26 ± 0.04
9.14	742	2-Pentenal	0.19 ± 0.06	20.29 ± 4.31	8.31 ± 1.68
10.48	777	3-Hexenal	3.14 ± 0.93	275.96 ± 42.84	270.77 ± 31.30
10.60	779	Hexanal	3.68 ± 0.86	192.41 ± 3.52	12.13 ± 1.27
12.40	837	(*E*)-2-Hexenal	25.72 ± 7.04	975.18 ± 40.25	332.79 ± 10.45
13.86	896	Heptanal	N.D.	1.13 ± 0.02	0.22 ± 0.11
14.14	908	(*E*,*E*)-2,4-Hexadienal	N.D.	48.24 ± 0.89	11.00 ± 1.45
16.08	967	Benzaldehyde	N.D.	0.65 ± 0.10	0.49 ± 0.05
17.64	1013	(*E*,*E*)-2,4-Heptadienal	0.09 ± 0.01	2.53 ± 0.26	1.32 ± 0.14
18.80	1049	Benzeneacetaldehyde	N.D.	0.77 ± 0.18	0.66 ± 0.06
20.62	1106	Nonanal	0.12 ± 0.02	0.42 ± 0.03	0.63 ± 0.04
**Ketones**					
4.78		2-Propanone	0.22 ± 0.08	1.08 ± 0.08	5.60 ± 0.82
5.65	587	2,3-Butanedione	N.D.	0.66 ± 0.16	0.65 ± 0.10
7.32	683	1-Penten-3-one	0.82 ± 0.04	85.48 ± 10.26	28.95 ± 6.68
7.55	696	3-Pentanone	N.D.	128.27 ± 20.89	2.02 ± 0.44
16.42	978	1-Octen-3-one	3.02 ± 1.22	84.21 ± 38.37	87.53 ± 10.67
20.29		3,5-Octadien-2-one	N.D.	0.23 ± 0.05	N.D.
**Terpenoids**					
5.02	508	Isoprene	N.D.	0.65 ± 0.15	0.34 ± 0.04
18.31	1034	(+)-(R)-Limonene	N.D.	N.D.	0.34 ± 0.01
19.66	1075	*cis*-Linalool oxide	N.D.	N.D.	0.47 ± 0.07
20.48	1100	β-Linalool	N.D.	N.D.	0.51 ± 0.01
3.22	1189	*p*-Menth-1-en-4-ol	N.D.	N.D.	0.66 ± 0.06
23.62	1203	α-Terpinol	N.D.	N.D.	0.13 ± 0.01
24.38	1229	β-Cyclocitral	0.13 ± 0.05	0.43 ± 0.07	0.20 ± 0.03
28.83	1388	β-Damascenone	0.11 ± 0.03	N.D.	0.29 ± 0.01
**Others**					
5.82	598	2-Methylfuran	N.D.	0.45 ± 0.25	N.D.
7.66	701	2-Ethylfuran	1.00 ± 0.44	109.48 ± 8.14	6.52 ± 0.59
12.76	893	3-Ethylthiophene	0.28 ± 0.09	199.94 ± 25.38	4.12 ± 0.54
15.91	960	5-Ethyl-2(5*H*)-furanone	N.D.	34.15 ± 6.35	25.34 ± 4.38
17.31	1003	3-Hexen-1-ol acetate	0.36 ± 0.01	3.67 ± 0.15	1.96 ± 0.20
24.19	1222	Ionene	N.D.	N.D.	0.09 ± 0.01
31.42	1488	β-Ionone	1.51 ± 0.47	0.78 ± 0.17	1.14 ± 0.16
Total known metabolites			19	32	38
Total volatile metabolites			48 ± 3	99 ± 8	97 ± 5

*^a^* Retention time (min). *^b^* Retention index. *^c^* Mean area (×10^6^) ± S.D. of three replicates; N.D., not detected.

## Data Availability

The data presented in this study are available in Supplementary Materials.

## Supplementary Materials

The following are available online at https://www.mdpi.com/article/10.3390/molecules26216473/s1, Table S1: Volatile metabolite profiles of glandular trichomes isolated from stems, trichome-free stems, and leaves using DVB/CAR/PDMS fiber at 60 °C and an extraction time of 20 min., Table S2: Loadings of volatile metabolites in the first two principal components (PC 1 and PC 2), and the site of origin.

## Sample Availability

Samples of the compounds are available from the authors.
