# Supplementary material for: An Optimized SPME-GC-MS Method for Volatile Metabolite Profiling of Different Alfalfa (Medicago sativa L.) Tissues
Source: Molecules. 2021 Oct 27;26(21):6473. doi: 10.3390/molecules26216473 (PMC8587762; doi:10.3390/molecules26216473)
Supplement: Supplementary file 1 [file molecules-26-06473-s001.zip › molecules-1418379-supplementary.pdf]

molecules

An optimized SPME-GC-MS method for volatile metabolite profiling of different alfalfa tissues

Dong Sik Yang [1,a,†], Zhentian Lei [2,†], Mohamed Bedair [3] and Lloyd W. Sumner [2,*]

[1] The Samuel Roberts Noble Foundation, 2510 Sam Noble Parkway, Ardmore, OK 73401, USA dongsiki@hanmail.net
[2] Metabolomics Center & Department of Biochemistry, University of Missouri, Columbia, MO 65211, USA; leiz@missouri.edu
[3] Bayer CropScience, 700 Chesterfield Parkway, West Chesterfield, MO 63017, USA; mohamed.bedair@bayer.com
[a] Current address: Samsung Particulate Matter Research Institute, Samsung Advanced Institute of Technology, 130 Samsung-ro, Yeongtong-gu, Suwon-si, Gyeonggi-do, Republic of Korea; dongsiki@hanmail.net
* Correspondence: Lloyd W. Sumner; sumnerlw@missouri.edu
† These authors contributed equally to this work.

Table Contents:

Table S1. Volatile metabolite profiles of glandular trichomes isolated from stems, trichome-free stems, and leaves using DVB/CAR/PDMS fiber at 60 °C and an extraction time of 20 min

Table S2. Loadings of volatile metabolites in the first two principal components (PC 1 and PC 2), and the site of origin

Supplemental Table S1

Volatile metabolite profiles of glandular trichomes isolated from stems, trichome-free stems, and leaves using DVB/CAR/PDMS fiber at 60 °C and an extraction time of 20 min.

No.	R.T.[a]	R.I.[b]	Compound	Ion[c] (m/z)	ID[c]	Trichome	Stem	Leaf
1	4.27		Sulfur dioxide	48.0	C	N.D.	0.73 ± 0.48[d]	8.90 ± 1.31
2	4.36		Acetaldehyde	43.2	C	N.D.	1.17 ± 1.16	10.00 ± 2.36
3	4.47		Methanethiol	48.2	C	N.D.	N.D.	0.39 ± 0.41
4	4.58		Ethyl alcohol	45.0	C	10.37 ± 1.51	N.D.	0.59 ± 0.26
5	4.75		2-Propenal	56.0	C	3.21 ± 0.33	3.66 ± 1.43	18.71 ± 5.11
6	4.77		2-Propanone	43.0	C	166.59 ± 11.12	43.28 ±11.08	114.97 ±4 3.20
7	4.79		Unknown	57.0		2.44 ± 0.20	N.D.	6.61 ± 1.87
8	4.82		Unknown	68.1		N.D.	N.D.	1.30 ± 0.31
9	5.01	508	Isoprene	59.0	A	1.27 ± 0.31	N.D.	4.06 ± 1.89
10	5.43	554	Unknown	41.0		1.53 ± 0.12	N.D.	2.59 ± 0.25
11	5.54	565	Unknown	46.0		N.D.	N.D.	1.81 ± 2.19
12	5.66	587	2,3-Butanedione	43.0	A	N.D.	N.D.	31.81 ± 2.75
13	5.72	595	Butanal	39.2	B	N.D.	N.D.	1.13 ± 0.12
14	5.75	597	2-Methylpropanal	43.0	B	6.93 ± 0.76	N.D.	N.D.
15	5.97	606	Unknown	43.0		3.96 ± 0.43	2.28 ± 0.29	7.37 ± 1.31
16	6.16	617	Unknown	35.0		N.D.	0.84 ± 0.39	N.D.
17	6.66	645	2-Butenal	39.0	B	N.D.	N.D.	10.18 ± 2.24
18	6.85	656	1-Butanol	56.0	A	1.82 ± 0.10	N.D.	N.D.
19	7.27	680	1-Penten-3-ol	57.0	A	7.28 ± 1.69	7.48 ± 2.94	104.81 ±17.79
20	7.32	683	1-Penten-3-one	55.0	A	12.38 ± 2.94	15.10 ± 6.97	484.09 ± 109.22
21	7.55	696	3-Pentanone	57.0	A	N.D.	5.20 ± 0.76	54.05 ± 9.43
22	7.62	700	Pentanal	41.0	A	N.D.	12.29 ± 0.96	17.02 ± 1.12
23	7.65	701	2-Ethylfuran	81.1	A	N.D.	N.D.	115.35 ± 35.74
24	8.54	725	3-Methyl-1-butanol	55.0	A	1.09 ± 0.16	2.04 ± 0.93	1.44 ± 0.44
25	8.67	729	3-Penten-2-one	68.9	B	N.D.	N.D.	2.36 ± 0.66
26	9.13	742	2-Pentenal	55.0	B	5.30 ± 0.39	4.39 ± 1.99	200.84 ± 54.35
27	9.43	750	1-Pentanol	70.1	A	0.86 ± 0.16	0.67 ± 0.15	11.09 ± 2.40
28	9.44	750	Unknown	55.0		N.D.	N.D.	15.63 ± 2.43
29	9.50	752	(*Z*)-2-Penten-1-ol	57.0	A	N.D.	2.02 ± 0.83	42.04 ± 10.13
30	10.15	770	Unknown	41.2		N.D.	N.D.	0.69 ± 0.16
31	10.41	777	3-Hexenal	41.0	B	72.25 ± 8.68	48.32 ±15.69	1350.94 ± 549.02
32	10.49	779	Hexanal	44.0	A	46.09 ± 6.42	70.75 ± 23.64	183.93 ± 27.14
33	10.85	789	Butyl acetate	208.9	A	19.08 ± 2.91	N.D.	N.D.
34	11.96	827	Unknown	39.0		1.64 ± 0.17	6.12 ± 2.65	111.49 ± 36.57

35	12.13	833	Methoxycarbonyl isothiocyanate	58.0	B	0.60 ± 0.12	N.D.	N.D.
36	12.23	837	(*E*)-2-Hexenal	39.0	A	41.20 ± 7.90	270.85 ±94.95	2310.32 ± 927.71
37	12.68	854	1-Hexanol	41.2	A	0.67 ± 0.20	N.D.	29.33 ± 12.68
38	12.70	854	Unknown	41.0		1.65 ± 0.19	N.D.	N.D.
39	12.74	856	2-Ethylthiophene	96.9	A	N.D.	N.D.	25.94 ± 9.29
40	13.76	893	Unknown	55.0		N.D.	N.D.	4.53 ± 0.78
41	13.84	896	Heptanal	39.0	A	1.55 ± 0.11	0.98 ± 0.61	8.56 ± 1.38
42	14.21	908	(*E*,*E*)-2,4-Hexadienal	81.2	A	3.64 ± 0.65	10.46 ± 5.42	136.46 ± 41.85
43	15.11	937	Unknown	57.0		0.51 ± 0.04	N.D.	N.D.
44	15.74	957	(*E*)-2-Heptenal	57.0	A	2.52 ± 0.27	1.60 ± 0.46	36.36 ± 10.86
45	15.84	960	5-Ethyl-2(5H)-furanone	55.0	B	12.58 ± 1.40	11.89 ± 6.65	333.45 ± 100.02
46	16.07	967	Benzaldehyde	77.1	A	1.09 ± 0.21	2.25 ± 0.95	8.37 ± 2.08
47	16.39	978	Hexanoic acid	60.0	A	N.D.	N.D.	30.89 ± 5.04
48	16.40	978	1-Octen-3-one	55.0	A	9.07 ± 1.13	N.D.	636.73 ± 276.21
49	16.41	978	Unknown	70.2		1.73 ± 0.15	N.D.	N.D.
50	16.49	981	1-Octen-3-ol	57.0	A	39.62 ± 6.10	61.96 ±21.35	3029.29 ± 1162.16
51	16.64	985	Unknown	43.0		11.28 ± 2.03	N.D.	N.D.
52	16.83	991	Myrcene	41.0	A	N.D.	2.48 ± 1.23	N.D.
53	16.87	993	2-Pentylfuran	81.1	A	1.82 ± 0.31	2.00 ± 1.17	7.34 ± 1.25
54	16.92	994	Unknown	67.1		N.D.	1.05 ± 0.44	N.D.
55	16.96	995	Unknown	55.0		N.D.	N.D.	8.21 ± 2.77
56	17.10	1000	Unknown	81.2		0.57 ± 0.10	0.99 ± 0.37	N.D.
57	17.30	1003	(*Z*)-3-Hexenyl acetate	67.1	A	N.D.	N.D.	23.29 ± 4.69
58	17.30	1003	Octanal	41.2	A	2.21 ± 0.52	0.63 ± 0.61	45.70 ± 8.75
59	17.42	1007	Unknown	67.2		1.30 ± 0.15	0.74 ± 0.56	N.D.
60	17.50	1010	(*E*,*E*)-2,6-Dimethyl-1,3,5,7-octatetraene	119.1	B	N.D.	N.D.	7.40 ± 2.13
61	17.60	1013	(*E*,*E*)-2,4-Heptadienal	81.1	A	0.85 ± 0.15	1.88 ± 0.70	39.32 ± 12.55
62	17.89	1022	Unknown	121.1		0.17 ± 0.03	N.D.	1.21 ± 0.50
63	18.07	1027	Unknown	69.1		0.91 ± 0.47	N.D.	N.D.
64	18.14	1029	*p*-Cymene	119.1	A	1.08 ± 0.06	1.95 ± 1.02	N.D.
65	18.17	1030	Unknown	91.1		N.D.	N.D.	29.49 ± 6.59
66	18.31	1034	D-Limonene	92.9	A	0.56 ± 0.07	2.10 ± 0.94	2.37 ± 0.62
67	18.37	1036	Unknown	83.1		1.89 ± 0.25	3.13 ± 1.97	58.06 ± 16.45
68	18.54	1041	2,2,6-Trimethylcyclohexanone	41.0	B	N.D.	N.D.	6.98 ± 2.70
69	18.64	1044	Unknown	55.0		N.D.	N.D.	33.11 ± 21.90
70	18.72	1047	Unknown	92.9		N.D.	0.54 ± 0.11	N.D.
71	18.73	1047	Unknown	82.9		0.42 ± 0.09	N.D.	5.38 ± 1.28
72	18.78	1049	Benzeneacetaldehyde	91.1	A	N.D.	1.53 ± 1.00	20.52 ± 3.95
73	19.11	1059	Unknown	57.0		7.31 ± 0.77	11.27 ± 4.89	N.D.
74	19.14	1060	(*E*)-2-Octenal	39.0	A	4.79 ± 0.11	N.D.	8.41 ± 1.77
75	19.20	1062	Unknown	57.0		N.D.	N.D.	242.02 ± 107.68
76	19.41	1068	(*E*)-2-Octen-1-ol	41.2	A	N.D.	N.D.	6.32 ± 1.86

77	19.51	1071	1-Octanol	41.2	A	N.D.	N.D.	6.32 ± 1.86
78	19.64	1075	*cis*-Linalool oxide	59.0	B	2.25 ± 0.19	7.48 ± 4.02	13.55 ± 2.57
79	19.79	1080	Unknown	68.1		N.D.	N.D.	4.46 ± 2.02
80	20.14	1090	Unknown	43.0		1.35 ± 0.08	N.D.	9.17 ± 1.97
81	20.36	1097	Unknown	55.0		N.D.	N.D.	19.91 ± 4.54
82	20.46	1100	*β*-Linalool	41.0	A	10.85 ± 0.35	42.69 ±14.86	400.77 ± 93.37
83	20.63	1106	Nonanal	41.0	A	46.37 ± 3.89	46.55 ± 16.41	1.62 ± 0.81
84	21.03	1119	Unknown	71.1		N.D.	N.D.	2.16 ± 0.88
85	21.34	1129	Unknown	109.1		N.D.	N.D.	3.73 ± 1.33
86	21.79	1143	Unknown	134.1		N.D.	N.D.	2.90 ± 0.90
87	22.00	1150	4-Oxoisophorone	39.2	A	0.24 ± 0.04	N.D.	2.90 ± 0.69
88	22.16	1155	Unknown	41.0		1.08 ± 0.25	4.44 ± 6.36	3.13 ± 2.31
89	22.37	1162	(*E*)-2-Nonenal	43.2	A	1.27 ± 0.07	6.80 ± 5.92	19.06 ± 2.83
90	22.39	1163	Octanoic Acid	60.0	A	3.76 ± 0.17	6.38 ± 6.02	N.D.
91	22.50	1166	Ocimenol	39.0	B	N.D.	4.22 ± 3.61	N.D.
92	22.50	1166	2-Nonen-1-ol	81.2	A	0.53 ± 0.08	1.07 ± 1.00	N.D.
93	22.65	1169	Unknown	41.0		2.38 ± 1.30	N.D.	N.D.
94	23.21	1189	*p*-Menth-1-en-4-ol	71.1	A	0.39 ± 0.06	0.61 ± 0.34	4.81 ± 1.37
95	23.32	1193	Unknown	43.0		N.D.	N.D.	4.75 ± 0.96
96	23.62	1203	*α*-Terpinol	59.0	B	19.61 ± 0.96	74.87 ±34.78	30.17 ± 6.70
97	23.74	1207	Decanal	41.0	A	11.75 ± 4.93	28.63 ±13.18	11.92 ± 2.44
98	24.10	1219	(*E*,*E*)-2,4-Nonadienal	81.1	A	0.60 ± 0.05	N.D.	2.23 ± 0.59
99	24.19	1222	Ionene	159.1	B	N.D.	N.D.	4.73 ± 0.87
100	24.31	1227	Unknown	94.2		2.38 ± 0.53	6.27 ± 3.34	4.57 ± 1.06
101	24.38	1229	Unknown	152.1		N.D.	N.D.	2.41 ± 0.50
102	24.89	1246	4-Oxononanal	55.0	B	1.65 ± 0.23	N.D.	2.87 ± 0.66
103	25.02	1251	Unknown	41.0		N.D.	5.78 ± 2.12	N.D.
104	25.31	1261	Nonanoic acid	45.0	A	3.16 ± 0.05	3.81 ± 4.55	18.40 ± 5.24
105	25.41	1264	(*E*)-2-Decenal	41.0	A	7.47 ± 0.36	N.D.	20.41 ± 4.56
106	26.37	1298	Unknown	81.2		0.39 ± 0.07	N.D.	N.D.
107	26.52	1303	N,N-Dibutylformamide	72.1	A	5.17 ± 0.44	N.D.	N.D.
108	27.66	1345	Unknown	84.1		0.22 ± 0.02	N.D.	N.D.
109	27.99	1357	Decanoic acid	41.0	A	2.77 ± 0.69	3.30 ± 2.73	3.14 ± 0.93
110	28.26	1367	Unknown	41.2		0.34 ± 0.05	N.D.	3.63 ± 0.86
111	28.37	1371	Unknown	141.1		N.D.	1.29 ± 1.24	N.D.
112	28.46	1374	Unknown	43.0		1.01 ± 0.12	1.58 ± 1.12	N.D.
113	28.60	1379	Unknown	70.9		0.40 ± 0.08	N.D.	0.01 ± 0.00
114	28.83	1388	*β*-Damascenone	69.1	B	N.D.	N.D.	11.48 ± 2.01
115	29.44	1411	Z-2-Dodecenol	41.2	B	N.D.	0.53 ± 0.57	N.D.
116	29.45	1411	Unknown	41.0		1.77 ± 1.65	N.D.	N.D.
117	30.43	1449	*cis*-Geranylacetone	43.0	B	2.99 ± 3.77	N.D.	3.14 ± 0.65
118	31.41	1488	β-Ionone	177.1	A	0.50 ± 0.15	2.34 ± 1.21	16.20 ± 5.73

119	31.71	1500	Unknown	43.0		0.38 ± 0.15	N.D.	N.D.
120	32.05	1513	Unknown	41.2		N.D.	0.36 ± 0.29	N.D.
121	34.14	1599	Unknown	57.2		0.22 ± 0.15	N.D.	2.82 ± 0.73
122	36.12	1686	Unknown	55.2		0.31 ± 0.13	N.D.	N.D.
123	36.44	1700	Unknown	43.2		0.27 ± 0.21	N.D.	3.24 ± 0.91
124	37.39	1744	Unknown	189.1		0.57 ± 0.09	N.D.	1.60 ± 0.36
125	38.35	1788	Unknown	57.0		0.97 ± 0.17	N.D.	1.74 ± 0.73
126	38.66	1802	Unknown	92.9		0.47 ± 0.06	N.D.	1.26 ± 0.53
127	39.29	1832	Unknown	143.1		0.93 ± 0.20	N.D.	3.61 ± 0.82
128	39.50	1843	6,10,14-Trimethyl-2-pentadecanone	43.2	B	0.65 ± 0.35	0.56 ± 0.66	4.83 ± 1.97
129	39.51	1843	Unknown	43.0		3.36 ± 3.29	N.D.	N.D.
130	39.73	1854	Unknown	227.1		1.01 ± 0.19	N.D.	3.13 ± 0.56
131	39.82	1858	Unknown	270.1		3.02 ± 0.51	0.51 ± 0.47	4.97 ± 0.88
132	40.74	1902	Unknown	157.1		0.76 ± 0.17	N.D.	2.37 ± 0.72
133	40.89	1910	Unknown	145.1		1.51 ± 0.31	N.D.	N.D.

[a] Retention time (min). [b] Retention index. [c] Fragment ion (m/z).

[c] Methods of identification: A, comparison of RI and mass spectra of identified compounds with those of an authentic compounds; B, tentatively comparison of mass spectrum with the NIST08/Wiley7 mass spectral library and RI with those from the literature; C, tentatively comparison of mass spectrum with the NIST08/Wiley7 mass spectral library.

[d] Relative concentration: 3-Pentanol equivalent (μg g^{-1}); Mean ± S.D. of four replicates; N.D., not detected.

Supplemental Table S2

Loadings of volatile metabolites in the first two principal components (PC 1 and PC 2), and the site of origin.

No.	Compound	PC1	PC2	Site of origin
001	Sulfur dioxide	0.321704	-0.04187	Leaf
002	Acetaldehyde	0.309259	-0.04825	Leaf
003	Methanethiol	0.241419	-0.00446	Leaf
004	Ethyl alcohol	-0.14265	0.368867	Glandular trichome
005	2-Propenal	0.319676	-0.02493	Leaf
006	2-Propanone	0.002692	0.370414	Glandular trichome
007	Unknown	0.301102	0.140167	Leaf
008	Unknown	0.319378	-0.00272	Leaf
009	Isoprene	0.277676	0.10666	Leaf
010	Unknown	0.258615	0.245266	Leaf
011	Unknown	0.199354	0.001185	Leaf
012	2,3-Butanedione	0.314697	-0.00284	Leaf
013	Butanal	0.310288	-0.00229	Leaf
014	2-Methylpropanal	-0.15851	0.361958	Glandular trichome
015	Unknown	0.292364	0.121477	Leaf
016	Unknown	-0.14447	-0.35561	Stem
017	(*E*)-2-Butenal	0.323546	-0.00403	Leaf
018	1-Butanol	-0.15879	0.362507	Glandular trichome
019	1-Penten-3-ol	0.324482	-0.0068	Leaf
020	1-Penten-3-one	0.322555	-0.0076	Leaf
021	3-Pentanone	0.318756	-0.039	Leaf
022	Pentanal	0.228894	-0.29817	Leaf
023	2-Ethylfuran	0.323027	-0.00385	Leaf
024	3-Methyl-1-butanol	-0.00772	-0.284	
025	3-Penten-2-one	0.319176	-0.00438	Leaf
026	(*E*)-2-Pentenal	0.321418	-0.00377	Leaf
027	1-Pentanol	0.32475	0.000164	Leaf
028	Unknown	0.324804	-0.00364	Leaf
029	(*Z*)-2-Penten-1-ol	0.323689	-0.02283	Leaf
030	Unknown	0.319234	-0.00336	Leaf
031	(*Z*)-3-Hexenal	0.314547	0.000766	Leaf
032	Hexanal	0.311825	-0.09369	Leaf
033	Butyl acetate	-0.15749	0.359835	Glandular trichome
034	Unknown	0.321498	-0.02103	Leaf
035	Methoxycarbonyl isothiocyanate	-0.15526	0.351409	Glandular trichome
036	(*E*)-2-Hexenal	0.312448	-0.04388	Leaf
037	1-Hexanol	0.314852	0.002953	Leaf

038	Unknown	-0.15839	0.36173	Glandular trichome
039	2-Ethylthiophene	0.320817	-0.00413	Leaf
040	Unknown	0.325182	-0.00357	Leaf
041	Heptanal	0.321405	0.014727	Leaf
042	(*E*,*E*)-2,4-Hexadienal	0.315034	-0.02702	Leaf
043	Unknown	-0.15846	0.362166	Glandular trichome
044	(*E*)-2-Heptenal	0.321126	0.003435	Leaf
045	5-Ethyl-2(5H)-furanone	0.32108	-0.00622	Leaf
046	Benzaldehyde	0.309527	-0.07433	Leaf
047	Hexanoic acid	0.322749	-0.00375	Leaf
048	1-Octen-3-one	0.316293	0.000237	Leaf
049	Unknown	-0.15879	0.360926	Glandular trichome
050	1-Octen-3-ol	0.318797	-0.00769	Leaf
051	Unknown	-0.15629	0.354823	Glandular trichome
052	Myrcene	-0.14457	-0.37464	Stem
053	2-Pentylfuran	0.31416	-0.03937	Leaf
054	Unknown	-0.14923	-0.37881	Stem
055	Unknown	0.319413	-0.0032	Leaf
056	Unknown	-0.26536	-0.19104	Stem
057	(*Z*)-3-Hexenyl acetate	0.323978	-0.00315	Leaf
058	Octanal	0.32386	0.007708	Leaf
059	Unknown	-0.25497	0.102515	
060	(*E*,*E*)-2,6-Dimethyl-1,3,5,7-octatetraene	0.31963	-0.003	Leaf
061	(*E*,*E*)-2,4-Heptadienal	0.321254	-0.01512	Leaf
062	Unknown	0.309243	0.047424	Leaf
063	Unknown	-0.13932	0.322711	Glandular trichome
064	*p*-Cymene	-0.24304	-0.22306	Stem
065	Unknown	0.325321	-0.00365	Leaf
066	D-Limonene	0.164965	-0.32497	
067	Unknown	0.322604	-0.01619	Leaf
068	2,2,6-Trimethylcyclohexanone	0.318868	-0.00443	Leaf
069	Unknown	0.260786	-0.00462	Leaf
070	Unknown	-0.15514	-0.37611	Stem
071	Unknown	0.323584	0.022974	Leaf
072	Benzeneacetaldehyde	0.32334	-0.03607	Leaf
073	Unknown	-0.26841	-0.18235	Stem
074	(*E*)-2-Octenal	0.26584	0.226652	Leaf
075	Unknown	0.304073	-0.00414	Leaf
076	(*E*)-2-Octen-1-ol	0.321608	-0.00328	Leaf
077	1-Octanol	0.321608	-0.00328	Leaf
078	*cis*-Linalool oxide	0.263607	-0.22371	Leaf
079	Unknown	0.305366	-0.00351	Leaf

080	Unknown	0.321595	0.051564	Leaf
081	Unknown	0.32443	-0.00344	Leaf
082	*β*-Linalool	0.314798	-0.03689	Leaf
083	Nonanal	-0.29876	-0.04265	
084	Unknown	0.318545	-0.00427	Leaf
085	Unknown	0.304366	-0.00425	Leaf
086	Unknown	0.312847	-0.00228	Leaf
087	4-Oxoisophorone	0.322443	0.027264	Leaf
088	Unknown	0.015917	-0.248	
089	(*E*)-2-Nonenal	0.283984	-0.16369	Leaf
090	Octanoic Acid	-0.19687	-0.20197	Stem
091	Ocimenol	-0.12347	-0.34843	Stem
092	2-Nonen-1-ol	-0.18039	-0.20721	Stem
093	Unknown	-0.13778	0.317685	Glandular trichome
094	*p*-Menth-1-en-4-ol	0.319722	-0.0323	Leaf
095	Unknown	0.32468	-0.00356	Leaf
096	*α*-Terpinol	-0.08439	-0.38897	Stem
097	Decanal	-0.11453	-0.3457	Stem
098	(*E*,*E*)-2,4-Nonadienal	0.304257	0.096594	Leaf
099	Ionene	0.322803	-0.00384	Leaf
100	Unknown	0.019244	-0.36877	
101	Unknown	0.32197	-0.00285	Leaf
102	4-Oxononanal	0.267939	0.220808	Leaf
103	Unknown	-0.15213	-0.38107	Stem
104	Nonanoic acid	0.290592	-0.04749	Leaf
105	(*E*)-2-Decenal	0.299654	0.142506	Leaf
106	Unknown	-0.15577	0.354256	Glandular trichome
107	N,N-Dibutylformamide	-0.15864	0.36156	Glandular trichome
108	Unknown	-0.15936	0.363011	Glandular trichome
109	Decanoic acid	-0.00885	-0.17569	
110	Unknown	0.316411	0.028823	Leaf
111	Unknown	-0.11733	-0.34116	Stem
112	Unknown	-0.23017	-0.19207	Stem
113	Unknown	-0.15569	0.352895	Glandular trichome
114	*β*-Damascenone	0.323236	-0.00383	Leaf
115	Z-2-Dodecenol	-0.1117	-0.30068	Stem
116	Unknown	-0.11495	0.259921	Glandular trichome
117	*cis*-Geranylacetone	0.109866	0.219438	
118	β-Ionone	0.31435	-0.05266	Leaf
119	Unknown	-0.14862	0.339352	Glandular trichome
120	Unknown	-0.12385	-0.35126	Stem
121	Unknown	0.311312	0.024735	Leaf

122	Unknown	-0.14241	0.319406	Glandular trichome
123	Unknown	0.306461	0.029674	Leaf
124	Unknown	0.30311	0.137594	Leaf
125	Unknown	0.249592	0.207136	Leaf
126	Unknown	0.291887	0.133571	Leaf
127	Unknown	0.31429	0.098513	Leaf
128	6,10,14-Trimethyl-2-pentadecanone	0.285546	-0.00988	Leaf
129	Unknown	-0.11283	0.256567	Glandular trichome
130	Unknown	0.308143	0.124908	Leaf
131	Unknown	0.266135	0.2082	Leaf
132	Unknown	0.300965	0.117724	Leaf
133	Unknown	-0.1559	0.357552	Glandular trichome
